# Cosmc overexpression enhances malignancies in human colon cancer

**DOI:** 10.1111/jcmm.14740

**Published:** 2019-10-21

**Authors:** Tianbo Gao, Tan Du, Xin Hu, Xichen Dong, Lina Li, Yakun Wang, Jian Liu, Lijie Liu, Tao Gu, Tao Wen

**Affiliations:** ^1^ Department of Oncology Beijing Chao‐Yang Hospital, Capital Medical University Beijing China; ^2^ Medical Research Center, Beijing Chao‐Yang Hospital, Capital Medical University Beijing China; ^3^ Department of Oncology First Hospital of Qinhuangdao Qinhuangdao China

**Keywords:** colorectal cancer (CRC), Cosmc, epithelial‐mesenchymal transition (EMT), invasion, migration, O‐glycosylation

## Abstract

Cosmc is known as a T‐synthase‐specific molecular chaperone that plays a crucial role in the process of O‐glycosylation. Cosmc dysfunction leads to inactive T‐synthase and results in aberrant O‐glycosylation, which is associated with various tumour malignancies. However, it is unclear whether Cosmc has some other functions beyond its involvement in O‐glycosylation. In this study, we aimed to investigate the functional role of Cosmc in human colorectal cancer (CRC). We first assessed the expression levels of Cosmc in human CRC specimens and then forcedly expressed Cosmc in human CRC cell lines (HCT116, SW480) to examine its impact on cellular behaviours. The mechanisms for aberrant expression of Cosmc in CRC tissues and the altered behaviours of tumour cells were explored. It showed that the mRNA and protein levels of Cosmc were markedly elevated in human CRC specimens relative to normal colorectal tissues. The occurrence of endoplasmic reticulum (ER) stress may largely contribute to the increased Cosmc expression in cancer tissue and cells. Cosmc overexpression in CRC cells significantly promoted cell migration and invasion, which could be attributed to the activation of the epithelial‐mesenchymal transition (EMT) pathway rather than aberrant O‐glycosylation. These data indicate that Cosmc expression was elevated in human CRC possibly caused by ER stress, which further enhanced malignancies through the activation of EMT but independently of aberrant O‐glycosylation.

## 1 INTRODUCTION

Mucin‐type O‐glycosylation (hereafter referred to as O‐glycosylation) of many secretory and transmembrane proteins is closely associated with cell growth and development^1^, ^2^, ^3^; abnormal alterations in O‐glycosylation may cause a neoplastic transformation of human cells and are frequently observed in various human cancers including colorectal cancer (CRC).^4^, ^5^, ^6^ O‐glycosylation is controlled by Cosmc, an endoplasmic reticulum (ER)‐localized molecular chaperone that regulates the formation of active T‐synthase.^7^, ^8^ Loss of Cosmc results in inactive T‐synthase and aberrant O‐glycosylation, characterized by the expression of Tn antigen.^7^, ^8^ Accumulating evidence indicates that dysfunctions in Cosmc such as acquired gene mutations, deletion, or hypermethylation are the prevailing mechanisms responsible for the phenotypes of aberrant O‐glycosylation in many human diseases.^7^, ^9^, ^10^, ^11^, ^12^, ^13^, ^14^ Many in vitro experiments also showed that Cosmc knockdown promoted malignant behaviours in various types of cancer cells such as growth, adhesion, migration and invasion through a mechanism depending on O‐glycosylation. Transfection of *wild‐type* Cosmc restored O‐glycosylation and attenuated malignant behaviours in cells.^4^ However, some recently emerged conflicting findings indicated rather opposing roles of Cosmc. For instance, Huang et al reported that Cosmc overexpression promoted malignant behaviours in cancer cells through induction of aberrant O‐glycosylation, whereas Cosmc knockdown reduced these malignancies, which indeed contradicted most reports.^15^ Lee et al also showed that overexpression of Cosmc in human umbilical vein endothelial cells enhanced T‐synthase, Tn antigen expression and cell growth.^16^ Moreover, it is noticeable that in many human cancers expression of Cosmc and Tn antigen is equally elevated,^17^ which is a paradox hard to explain by the theory of aberrant O‐glycosylation. It is unclear whether elevated Cosmc expression has additional effects or is just an outcome resulting from the increased Tn antigen via feedback regulation. So far, the pathological role of Cosmc in human CRC remains largely elusive.

Here we first defined the expression of Cosmc in clinical CRC samples. We observed that Cosmc expression was up‐regulated at both mRNA and protein levels in cancer samples compared with normal colorectal tissues. By investigating the mechanisms of elevated Cosmc expression in human CRC tissues, we found that ER stress was likely an important cause for elevated Cosmc expression. We further assessed the functional impact of Cosmc by using Cosmc‐overexpressing CRC cell lines and found that Cosmc overexpression significantly promoted cell migration and invasion. Notably, Cosmc overexpression did not affect the process of O‐glycosylation, because there were no appreciable changes in Tn antigen, T‐synthase and/or ppGalNac‐Ts. Interestingly, we found that the epithelial‐mesenchymal transition (EMT) process was activated prominently in Cosmc‐overexpressing cells, which might be responsible for the altered oncogenic features in cells.

## 2 MATERIAL AND METHODS

### 2.1 Clinical specimens and cell lines

The tumour tissues were freshly acquired by surgical resection from 85 patients with CRC at Beijing Chao‐Yang Hospital, Capital Medical University, Beijing, China. Normal colorectal mucosae were taken at biopsy from 42 individuals without colorectal malignancies. The specimens were stored at −80°C for further analysis. Each patient provided written consent. The study was approved by the Ethics Committees of Beijing Chao‐Yang Hospital, Capital Medical University, which followed the recommendations of the Declaration of Helsinki for biomedical research involving human subjects.

Human colon cancer cell lines HCT116 and SW480 were obtained from the American Type Culture Collection (ATCC, USA) and cultured in Dulbecco's modified Eagle's medium (Sigma, USA) supplemented with 10% foetal bovine serum (Gibco, USA), 100 U/mL penicillin, and 100 μg/mL streptomycin. Cells were cultured in a humidified incubator with 5% CO_2_ at 37°C.

### 2.2 RNA extraction and real‐time RT‐PCR

Total RNA was isolated from frozen CRC tissues and cell lines using Trizol reagent (Invitrogen, Life Technologies, Carlsbad, CA), according to the manufacturer's instruction. The concentration and purity of RNA were measured using a NanoDrop ND‐2000 Spectrophotometer (Thermo Scientific Wilmington, DE, USA). 2 μg of RNA was reverse‐transcribed into cDNA using a reverse transcription system (TransGen Biotech, China). The Real‐time PCR analysis was conducted on the 7500 Sequence Detection System (Applied Biosystems, China) using SYBR Premix according to the manufacturer's protocol with GPADH as an internal control. The thermal cycling conditions were as follows: 2 seconds at 95°C, followed by 40 cycles of 95°C for 5 seconds and 60°C for 34 seconds. Relative fold changes were normalized with GAPDH and calculated using 2^‐ΔΔCT^ methods. For Cosmc mRNA detection, the forward primer was 5′‐ACTGCAGCCCAAAGACTCACATCT‐3′, and the reverse primer was 5′‐ATGCACCACCATGAGCATCATCAC‐3′. As for GAPDH detection, the forward and reverse primers were 5′‐AATCCCATCACCATCTTCCA‐3′ and 5′‐TGGACTCCACGACGTACTCA‐3′. The sequences of the primers for ppGalNAc‐Ts were shown in the supplementary data.

### 2.3 Western blot analysis

Frozen tissues or cells were lysed using RIPA lysis buffer (EnoGene, China) supplemented with 1 mM protease inhibitor cocktail (EnoGene, China) and 1 mM phenylmethylsulfonyl fluoride (PMSF, EnoGene, China). After centrifugation at 12 000× *g* for 20 minutes at 4°C, the supernatant was collected. The concentration of total proteins was measured with the BCA Protein Assay Kit (Thermo Fisher, USA). Samples containing an equal amount of denatured proteins were separated on a 10% SDS polyacrylamide gel and transferred onto a PVDF membrane (Millipore, USA). The membrane was blocked with 5% non‐fat milk for 1 hour at room temperature and probed with primary antibodies and HRP‐conjugated secondary antibodies. Signals were detected using a chemiluminescent HRP substrate (Millipore, USA) on the Bio‐Rad imaging system. The following primary antibodies were used: Cosmc (1:200, Santa Cruz), T‐synthase (1:200, Santa Cruz), GRP78 (1:1000, Cell Signaling Technology), CHOP (1:1000, Cell Signaling Technology), ZO‐1 (Zonula‐occludens‐1) (1:1000, Cell Signaling Technology), Vimentin (1:1000, Cell Signaling Technology), Slug (1:1000, Cell Signaling Technology), Snail (1:1000, Cell Signaling Technology) and GAPDH (1:1000, Cell Signaling Technology).

### 2.4 Lentivirus‐mediated Cosmc transfection

The Cosmc overexpression plasmid was constructed by cloning Cosmc CDS (F:5′‐GAGGATCCCCGGGTACCGGTCGCCACCATGCTTTCTGAAAGCAGCTCC‐3′; R:5′‐CACACATTCCACAGGCTAGTCAGTCATTGTCAGAACCATTTG‐3′) into the GV367‐EGFP lentiviral vector containing restriction sites for AgeI/ NheI, which was purchased from Shanghai Genechem Co. Ltd (China). The empty vector was used as the control. The Cosmc expression vector and the empty vector were transfected into HCT116 and SW480 cells with polybrene respectively, according to the manufacturer's instruction. The transfected cells were cultured for 72 hours and then selected with complete medium containing 2 μg/mL puromycin (Gibco, USA) for 7 days.

### 2.5 Transwell migration and invasion assays

After being starved for 24 hours, 2 × 10^5^ cells with the serum‐free medium were seeded into the upper chamber (8‐μm pore size, BD Bioscience, USA) pre‐coated with or without Matrigel (BD Bioscience, USA). Complete medium containing 10% FBS was added to the bottom of each well as a chemoattractant. After being cultured for 24 hours, non‐migrated or non‐invaded cells were removed off the upper chamber using a cotton swab while the migratory or invasive cells were counted after fixation with 4% paraformaldehyde and being stained with 0.1% crystal violet.

### 2.6 Flow cytometry analysis

To analyse the expression of Tn antigen in Cosmc‐overexpressing cells and control cells, flow cytometry analysis was performed. Briefly, 1 × 10^5^ cells were routinely trypsinized, suspended in cold PBS and then incubated with 10 μg/mL mouse anti‐Tn IgM mAb or mouse IgM isotype‐antibody as a control (Santa Cruz, USA) for 2 hours at 4°C.^4^ After being washed three times with PBS, cells were incubated with PE‐labelled goat antimouse IgM secondary antibody (BD, USA) for 1 hour at 4°C. Cells were washed three times and suspended in 500 μL cold PBS per tube, followed by further analysis on a Flow Cytometer (Canto II, BD Bioscience, USA).

### 2.7 Statistical analysis

All data were analysed with GraphPad Prism 6.0 (GraphPad Software, La Jolla, CA, USA). All experiments were repeated at least in triplicate, and the data were presented as mean ± standard deviation (SD). Differences were analysed by Student's *t* test (unpaired, 2‐tailed), and *P* < .05 was considered statistically significant.

## 3 RESULTS

### 3.1 Cosmc expression was up‐regulated in human CRC tissues

**Figure 1:**
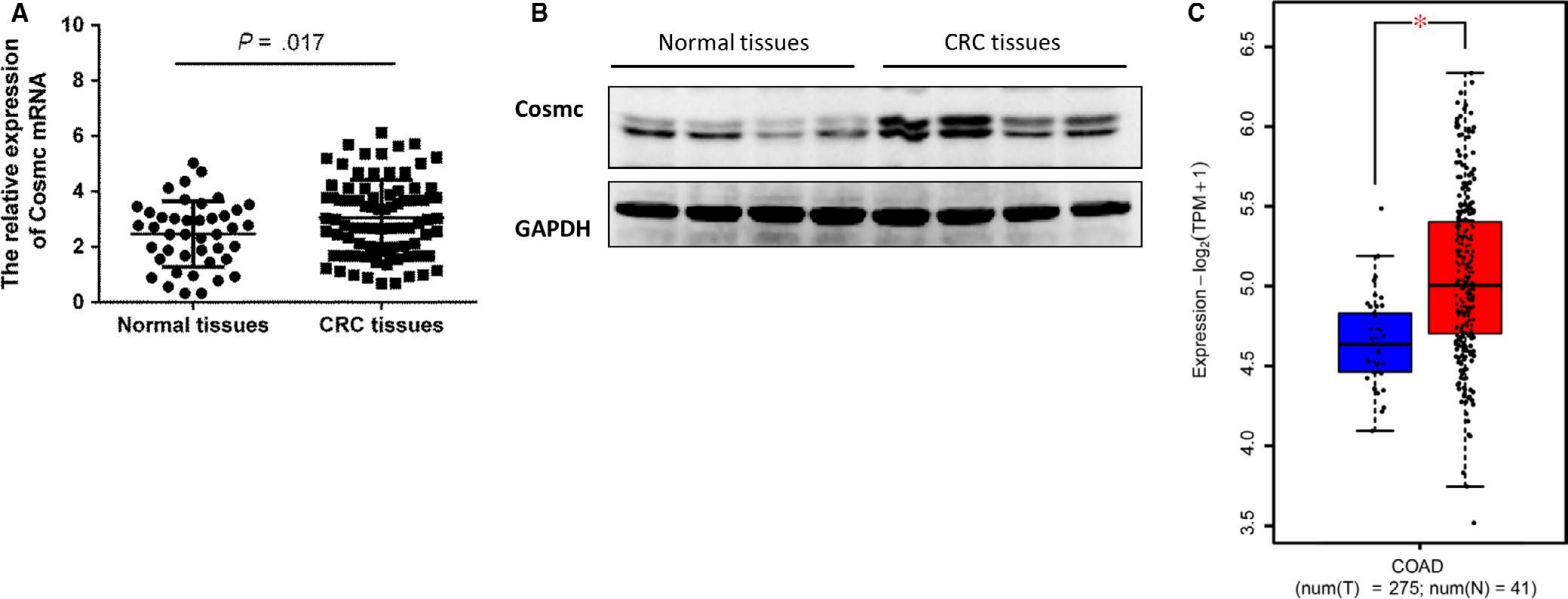
Cosmc expression is elevated in human CRC. A, The mRNA levels of Cosmc were measured by quantitative real‐time RT‐PCR. It showed that Cosmc mRNA was significantly higher in CRC tissues (n = 85) than normal colorectal tissues (n = 42) (*P* = .017). B, The protein levels of Cosmc were shown to be elevated in human CRC tissues when compared with normal controls. A representative blot from 4 samples of CRC tissues and 4 samples of normal subjects was shown. Elevated. C, TCGA database analysis showing that Cosmc mRNA expression was increased in human CRC tissues (n = 275) in contrast to normal controls (n = 41)

Cosmc is an essential molecular chaperone for T‐synthase and dysfunction of Cosmc results in abnormal exposure of Tn antigen, which is recognized as a hallmark for aberrant O‐glycosylation.^8^, ^9^ However, the precise role of Cosmc in clinical CRC remains largely unexplored. To determine whether there are aberrant alterations in Cosmc expression in clinical CRC, we examined its mRNA and protein levels in archived frozen human colorectal tissues (42 normal colorectal tissues, 85 CRC tissues). Real‐time RT‐PCR and Western blot analysis showed that Cosmc mRNA and protein levels were both elevated in human CRC tissues when compared with normal colorectal tissues (both *P* < .01; Figure 1A,B). This study cannot assess the proper localization of Cosmc, because the anti‐Cosmc antibody is not suitable for immunohistochemistry. Besides, we analysed RNA‐seq data of TCGA colon adenocarcinoma (COAD) data set including 275 tumour and 41 adjacent normal samples. It exhibited significantly higher Cosmc mRNA levels in CRC patients in comparison with normal controls (*P* < .05) (Figure 1C), which is consistent with our observations.

### 3.2 ER stress contributed to elevated Cosmc expression in human CRC

**Figure 2:**
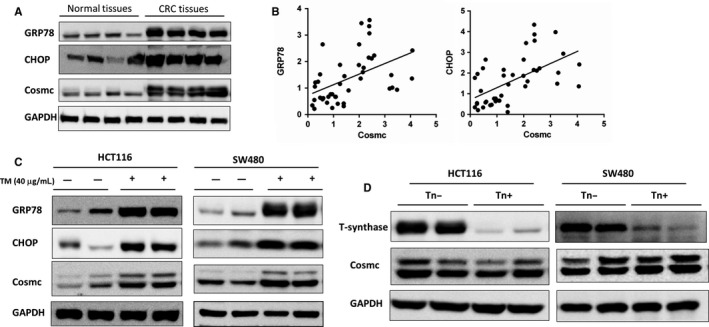
The mechanisms for elevated Cosmc expression in CRC. A, The protein levels of GRP78, CHOP and Cosmc were measured by Western blot. A representative blot from 4 samples of normal tissues and 4 samples of CRC tissues was shown. B, Significant correlations between Cosmc and GRP78, CHOP were observed. C, Western blot demonstrated that tunicamycin (TM, 40 μg/mL) treatment caused remarkable up‐regulation of GRP78, CHOP and Cosmc expression in HCT116 and SW480 cells, respectively. D, T‐synthase deficiency in HCT116 and SW480 cells caused no appreciable changes in Cosmc protein expression

Although loss of functional Cosmc caused by mutation, deletion, or hypermethylation has been widely recognized as a prevailing mechanism underlying aberrant O‐glycosylation observed in several types of cancers including CRC,^7^, ^9^, ^10^, ^11^, ^12^, ^13^, ^14^ little is known why there is an elevated expression of Cosmc in CRC, which appears to contradict the data obtained from some tumour‐derived cell lines and certain types of malignancies such as pancreatic cancer.^18^ Here we sought to investigate the molecular mechanisms underlying elevated Cosmc expression in CRC tissues. It has been proposed that expression of Cosmc may be induced by ER stress, which occurs frequently in malignancies.^17^ Moreover, Tn antigen has been discovered to be abundantly expressed in human CRC, which may promote Cosmc production via a feedback loop.^17^ To test these possibilities, we examined the expression levels of ER stress markers such as GRP78 and CHOP in another set of frozen CRC tissues (16 normal colorectal tissues, 32 CRC tissues). Western blot results showed that both GRP78 and CHOP were significantly up‐regulated in CRC tissues as compared with normal controls, indicative of the occurrence of ER stress, which also correlated with the expression of Cosmc (Figure 2A). Notably, there was a strong correlation between expression of Cosmc and ER stress in human CRC tissues (Figure 2B).

To definitively address this possibility, we treated two types of CRC cell lines (HCT116, SW480) with tunicamycin (TM, Sigma, MO, USA) to induce ER stress. At 24 hours, administration of TM (40 μg/mL) led to the occurrence of ER stress in both cell lines, as evidenced by a remarkable up‐regulation of GRP78 and CHOP protein expression (Figure 2C). It is noticeable that Cosmc was also significantly increased at its protein levels following ER stress, thereby supporting the likelihood that ER stress may induce Cosmc expression in cancer cells.

In addition, it has been confirmed that Cosmc dysfunction induces Tn antigen expression,^8^, ^9^ but it is uncertain whether the increased Tn antigen in turn promotes Cosmc production via a feedback loop. To address this question, we used Tn‐expressing CRC cell lines (HCT116, SW480), which were previously generated through forced deletion of the key glycosyltransferase for O‐glycosylation T‐synthase,^19^, ^20^ to assess whether high levels of Tn antigen have an impact on Cosmc expression. T‐synthase deficiency is sufficient to induce Tn antigen expression in cells (data not shown). In both cell lines, we observed no appreciable differences in the levels of Cosmc expression between Tn‐positive and Tn‐negative cells, suggesting that Tn antigen may have little influence on expression of Cosmc (Figure 2D). There may exist other possibilities contributing to Cosmc up‐regulation in CRC tissues, which deserves more future work.

### 3.3 The effects of Cosmc overexpression on CRC cell behaviours

**Figure 3:**
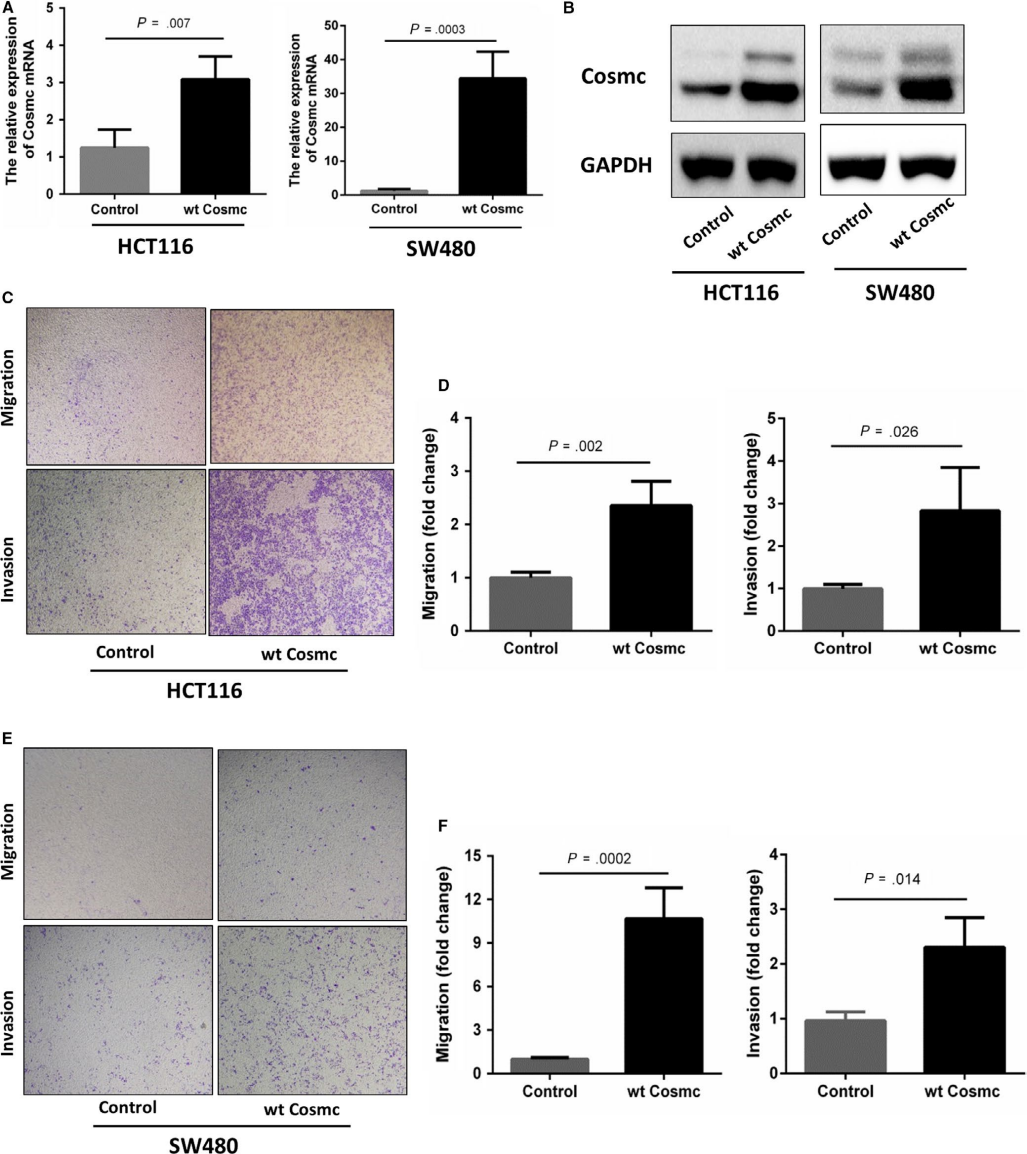
Cosmc overexpression promotes cellular migration and invasion in CRC cells. A, qRT‐PCR analysis of Cosmc mRNA levels in HCT116 and SW480 cells following Cosmc transfection. B, Western blot analysis of Cosmc overexpression in HCT116 and SW480 cells. C, Transwell migration and invasion assays in HCT116 cells overexpressing Cosmc and their corresponding control cells. D, Quantification of cell migration and invasion abilities in HCT 116 cells. E, The migration and invasion assays in SW480 cells overexpressing and their corresponding control cells. F, Quantification of cell migratory and invasive abilities in SW480 cells. Experiments were performed at least in triplicate, and representative images were shown

We next studied whether the elevated Cosmc expression influences CRC progression. Two types of human colon cancer cell lines HCT116 and SW480 were chosen to stably transfect with GV367‐Cosmc‐EGFP (containing Cosmc sequences) and GV367‐Control‐EGFP lentiviral vectors, respectively. The Cosmc overexpression in both cell lines was confirmed at both mRNA and protein levels. RT‐PCR and Western blot analysis showed that compared with the control cells, the mRNA and protein levels of Cosmc in HCT116 and SW480 cells were significantly higher than the controls (Figure 3A,B). We then performed transwell assays to examine cell migratory and invasive abilities in both cell lines. As shown in Figure 3C‐F, both Cosmc‐overexpressing cell lines exhibited a significant increase in their migratory and invasive abilities in comparison with their respective controls. These results provided evidence that Cosmc overexpression can promote malignant properties of colon cancer cells in vitro.

### 3.4 Cosmc overexpression‐mediated oncogenic alterations were not attributed to aberrant O‐glycosylation

**Figure 4:**
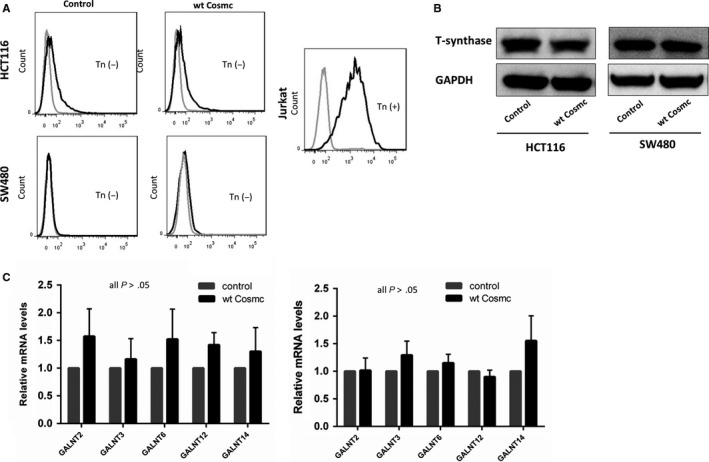
Cosmc overexpression is independent of aberrant O‐glycosylation. A, Flow cytometric analysis of Tn antigen expression in HCT116 and SW480 cells overexpressing Cosmc. Jurkat cells were included as the positive control. It showed that Cosmc overexpression caused no expression of Tn antigen in CRC cells. B, Western blot analysis of T‐synthase in HCT116 and SW480 cells showed no appreciable changes in the expression of T‐synthase following Cosmc overexpression. C, qRT‐PCR analysis of the mRNA levels of five ppGalNAc‐Ts, which are closely associated with CRC, in HCT116 and SW480 cells following Cosmc overexpression. Experiments were performed at least in triplicate, and representative images were shown

Most studies have confirmed that aberrant O‐glycosylation mediated by the loss of functional Cosmc contributes to tumour progression and metastasis in a broad range of human cancers including CRC.^4^, ^9^, ^18^ Here we questioned whether the tumour‐promoting effects of Cosmc overexpression were also due to aberrant O‐glycosylation. To address this issue, we examined the expression of Tn antigen, T‐synthase, and ppGalNac‐Ts, which are essential components during the process of O‐glycosylation, in CRC cells. Alterations in Tn antigen, T‐synthase and/or ppGalNac‐Ts are indicative of aberrant O‐glycosylation.^21^, ^22^, ^23^, ^24^ We first detected Tn antigen expression in both Cosmc‐overexpressing cells via flow cytometry. Jurkat cells that harbour mutations in Cosmc, thus expressing abundant Tn antigen, were included as a positive control. The results showed no staining of Tn antigen in both cells transfected with Cosmc and empty vector, respectively. Likewise, Western blot demonstrated that both Cosmc‐overexpressing cell lines had comparable levels of T‐synthase to the control cells, suggesting that Cosmc overexpression does not necessarily affect T‐synthase expression. In addition, ppGalNAc‐Ts catalyse the initial O‐glycosylation step, so the changes in the ppGalNAc‐Ts expression may represent alterations in the process of O‐glycosylation.^25^, ^26^ Here we used RT‐PCR to determine ppGalNac‐Ts' mRNA levels in Cosmc‐overexpressing cells. We selected 5 types of ppGalNac‐Ts (GALNT2, GALNT3, GALNT6, GALNT12, GALNT14), as these ppGalNac‐Ts have been reported to be closely associated with CRC.^27^, ^28^, ^29^, ^30^, ^31^ Our analysis did not detect obvious differences in the mRNA levels of these ppGalNAc‐Ts between Cosmc‐overexpressing cells and the control cells (Figure 4C). Collectively, these results suggested that aberrant O‐glycosylation is unlikely the cause for Cosmc overexpression‐mediated oncogenic alterations.

### 3.5 Cosmc overexpression activated the EMT signalling pathway

**Figure 5:**
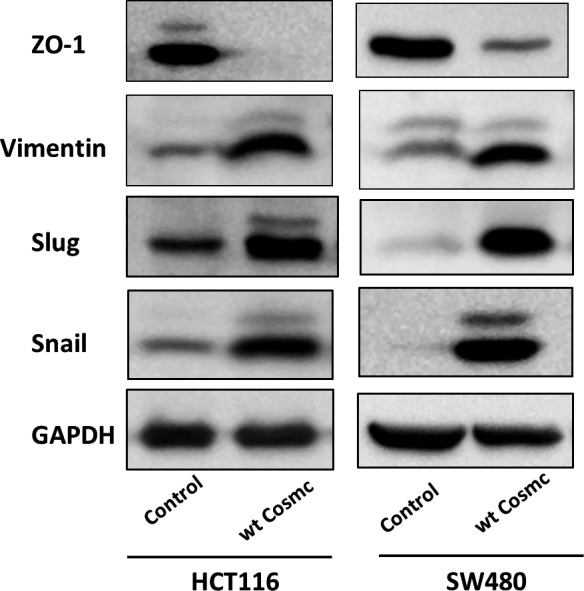
Cosmc overexpression activates the EMT process. Western blot analysis of the protein levels of ZO‐1, Vimentin, Slug and Snail in HCT116 and SW480 cells following Cosmc overexpression. Experiments were performed at least in triplicate, and representative images were shown

We further explored how Cosmc overexpression alters CRC cellular behaviours. The EMT process is an essential candidate signalling pathway associated with cancer progression and metastasis.^32^ We thereby analysed whether Cosmc overexpression can influence the EMT process. As shown in Figure 5, Cosmc overexpression induced typical characteristics of EMT in both cell lines, as evidenced by a decrease in the expression level of ZO‐1, a classical epithelial marker, along with an increase in the expression levels of several mesenchymal markers including Vimentin, Slug and Snail. These data supported that Cosmc overexpression can activate EMT, which may be a major mechanism responsible for enhanced malignancies in colon cancer cells.

## 4 DISCUSSION

The discovery of Cosmc was an important biological event in the research history of O‐glycosylation.^7^, ^8^, ^9^ More recent studies disclosed that Cosmc is a specific molecular chaperone for T‐synthase and Cosmc loss‐of‐function leads to impaired expression or function of T‐synthase, and subsequently an occurrence of aberrant O‐glycosylation, which is characterized by the expression of Tn antigen.^8^, ^9^, ^18^ Therefore, many studies investigated the functional role of aberrant O‐glycosylation in human diseases via manipulation of Cosmc. For instance, Hofmann et al analysed the impact of aberrant O‐glycosylation on pancreatic cancer cells through lentivirus‐mediated knockdown of Cosmc.^18^ Radhakrishnan et al also used gene editing to specifically knockout Cosmc in several pancreatic cancer cell lines, aiming to determining the biological and pathological role of aberrant O‐glycosylation in tumorigenesis.^33^ Most findings support that loss of Cosmc expression/function is a major cause for aberrant O‐glycosylation detected in various types of human cancers.^9^, ^18^, ^34^ However, recently some controversial reports regarding the expression of Cosmc in human tumours emerged. Huang et al reported that overexpression rather than the impaired expression of Cosmc enhanced malignant behaviours in colon cancer cells. They concluded that Cosmc overexpression increased T‐synthase expression, which further produced more O‐glycans such as T antigen to affect cellular behaviours.^15^ In their study, Cosmc knockdown was demonstrated to be able to decrease cancer cellular malignancies, which actually contradicted most recent findings. Thus, the role of Cosmc in addition to mediating O‐glycosylation needs to be fully explored.

In this study, we focused our attention on the impact of Cosmc overexpression in human colon cancer. We first assessed the expression levels of Cosmc in human CRC tissues. We found that Cosmc expression was up‐regulated in tumour tissues relative to normal colorectal tissues at its mRNA and protein levels. TCGA data set analysis also supported our observations. Because Cosmc loss‐of‐function is a prevailing mechanism for aberrant O‐glycosylation detected in many human cancers,^9^, ^18^, ^22^ this finding appeared paradoxical. We thereby sought to explore the mechanisms for elevated Cosmc expression in human CRC tissues. Sun et al suggested that ER stress, which occurs frequently in human malignancies, may induce Cosmc expression.^17^ In addition, the increased Tn antigen in CRC tissues may regulate Cosmc production through a feedback loop.^17^ To test these possibilities, we examined the occurrence of ER stress in our archived CRC samples. It showed that the expression of GRP78 and CHOP, the canonical markers for ER stress, were elevated in CRC tissues as compared with normal tissues. There was a clear correlation between ER stress and Cosmc expression in human specimens. We performed in vitro studies to further validate this observation and found that Cosmc expression was drastically up‐regulated following the induction of ER stress in cancer cells, thereby supporting that ER stress may contribute to the elevated Cosmc expression in human CRC. Meanwhile, we also checked the possibility of whether abundant expression of Tn antigen, which has been detected in human CRC, can in turn promote Cosmc production. The Tn‐expressing CRC cell lines, which were previously established by forced depletion of T‐synthase,^20^ did not exhibit an appreciable difference in Cosmc expression as compared with the control cells, thus ruling out this possibility. However, it should be noted that there may exist other mechanisms for elevated Cosmc expression in human CRC awaiting future consideration.

Although it has been confirmed that dysfunction in Cosmc promotes tumorigenesis via aberrant O‐glycosylation of many key glycoproteins,^3^, ^10^, ^34^, ^35^ little is known whether up‐regulation of Cosmc could affect tumour progression. We established two types of colon cancer cell lines (HCT116, SW480) stably overexpressing Cosmc to examine its functional impact. Compared with the controls, Cosmc overexpression significantly promoted oncogenic features such as cell migration and invasion in both cell lines. These findings provide evidence that Cosmc overexpression may also contribute to CRC progression and metastasis. In a word, both Cosmc overexpression and reduction have similarly tumour‐promoting effects. We questioned whether Cosmc overexpression may also affect the process of O‐glycosylation as Cosmc dysfunction does. Huang et al reported that Cosmc overexpression increased T‐synthase expression and T antigen production, which was the mechanism for the altered cellular phenotypes.^15^ Here we checked the changes of Tn, T‐synthase, and several essential ppGalNAc‐Ts, which are key components of normal O‐glycosylation,^21^, ^24^, ^26^ in both cell lines overexpressing Cosmc. However, we observed no comparable difference in the expression of Tn, T‐synthase and ppGalNAc‐Ts. These data suggested that overexpression of Cosmc does not necessarily cause aberrant O‐glycosylation. Cosmc may have other important functions in addition to involvement in O‐glycosylation. Because the EMT process is closely associated with cancer progression and metastasis,^32^, ^36^, ^37^ we reasoned if Cosmc acts upon EMT to affect cellular behaviours. Here we found that Cosmc overexpression significantly activated the EMT pathway. Although a classical epithelial marker E‐cadherin was not detected in both cell lines, another epithelial marker ZO‐1 was demonstrated to decrease markedly, accompanied by an enhanced expression of several mesenchymal cell markers, which represented sufficiently the characteristics of EMT.^32^, ^38^

In summary, our findings in human CRC specimens and cell lines indicated that Cosmc overexpression, which may be induced by ER stress, is not associated with elevated T‐synthase and Tn expression. Similar to Cosmc loss‐of‐function, overexpression of Cosmc can also promote malignancies (cell migration and invasion) in CRC cells but their individual mechanisms may differ. The biological effects of Cosmc loss‐of‐function are likely dependent on aberrant O‐glycosylation,^8^, ^9^, ^12^ which have been confirmed in many studies, whereas Cosmc overexpression may exert its effects possibly via activation of the EMT process. To our knowledge, it is the first to investigate the mechanisms and functions related to elevated Cosmc expression. Our results may explain why most of the Tn‐bearing CRC specimens have elevated Cosmc expression. It also suggests more complicated mechanisms for Tn expression in human cancers, which awaits further investigation.

## CONFLICT OF INTEREST

The authors confirm that there are no conflicts of interest.

## AUTHOR CONTRIBUTIONS

Tianbo Gao and Tan Du participated in the study design and performed some experiments and wrote the manuscript. Xin Hu and Xichen Dong performed some experiments. Lina Li and Yakun Wang contributed cellular models for this study. Jian Liu and Lijie Liu designed the research and analysed the data. Tao Gu and Tao Wen designed the study, interpreted the data and wrote the manuscript. All authors read and approved the final manuscript.

## Data Availability

The data that support the findings of this study are available from the corresponding author upon reasonable request.
