# Supplementary material for: Cosmc overexpression enhances malignancies in human colon cancer
Source: J Cell Mol Med. 2019 Oct 21;24(1):362–70. doi: 10.1111/jcmm.14740 (PMC6933370; doi:10.1111/jcmm.14740)
Supplement: Supplementary file 1 [file JCMM-24-362-s001.docx]

**Supporting information**

**Methods**

Total RNA was extracted from CRC cells using TRIzol reagent (Invitrogen, CA, USA), according to the manufacturer’s instructions. The RNA quality was assessed by a NanoDrop 2000 spectrophotometer (Wilmington, USA). ppGalNAc-Ts levels were measured by qPCR using SYBR Premix (Applied Biosystems) on the 7500 Sequence Detection System (Applied Biosystems). GAPDH was used as an internal control.

The sequences of all primers were as follows:

ppGalNAc-T2: F 5’- ACTACAGCAATGATCCTGAGG-3’

R 5’- TCGATGATGGGTGACACAACC-3’

ppGalNAc-T3: F 5’-ACACTCGACCTCCTGAATGTA-3’

R 5’-ATCATGTAAGTACTCATCTACACTAG-3’

ppGalNAc-T6: F 5’-CCAGCACAGAGGAGCACCTA-3’

R 5’- CGTGGAAGCACTCACAGTGG-3’

ppGalNAc-T12: F 5’- TGACTACAGTGATAGAGAGCACC-3’

R 5’- TCCTCTTCATGGATCCTCTGC-3’

ppGalNAc-T14: F 5’- AGGGTCAAAGAGGACTACACG-3’

R 5’- CCAGCTATGATAGGAGTCCTG-3’
